# Enhanced segmentation improves 4D blood flow quantification in patients with tetralogy of Fallot and pulmonary regurgitation

**DOI:** 10.1186/1532-429X-16-S1-P118

**Published:** 2014-01-16

**Authors:** Joshua D Robinson, Cynthia K Rigsby, Alex J Barker, Kelly B Jarvis, Roger A de Freitas, Susanne Schnell, Michael Markl

**Affiliations:** 1 Cardiology, Ann & Robert H Lurie Children's Hospital, Chicago, Illinois, USA; 2 Medical Imaging, Ann & Robert H Lurie Children's Hospital, Chicago, Illinois, USA; 3 Northwestern University, Chicago, Illinois, USA

## Background

2D phase contrast (PC) MRI provides reliable quantification of blood flow in pts with tetralogy of Fallot (TOF). While 2D PC is the standard for evaluating pulmonary regurgitant fraction, it is limited by single direction velocity measurement and may be inadequate to characterize 3D hemodynamics. Multiple planar acquisitions are often required. While 4D flow MRI provides simultaneous assessment of 3D flow characteristics of all vessels within a volume and offers the ability to retrospectively quantify blood flow parameters at selectable regions of interest, these exams require substantial post-processing and adjacent structures may be difficult to analyze. We compared traditional 2D PC and 4D flow quantification in patients with TOF using both traditional and enhanced segmentation techniques.

## Methods

**Figure 1:**
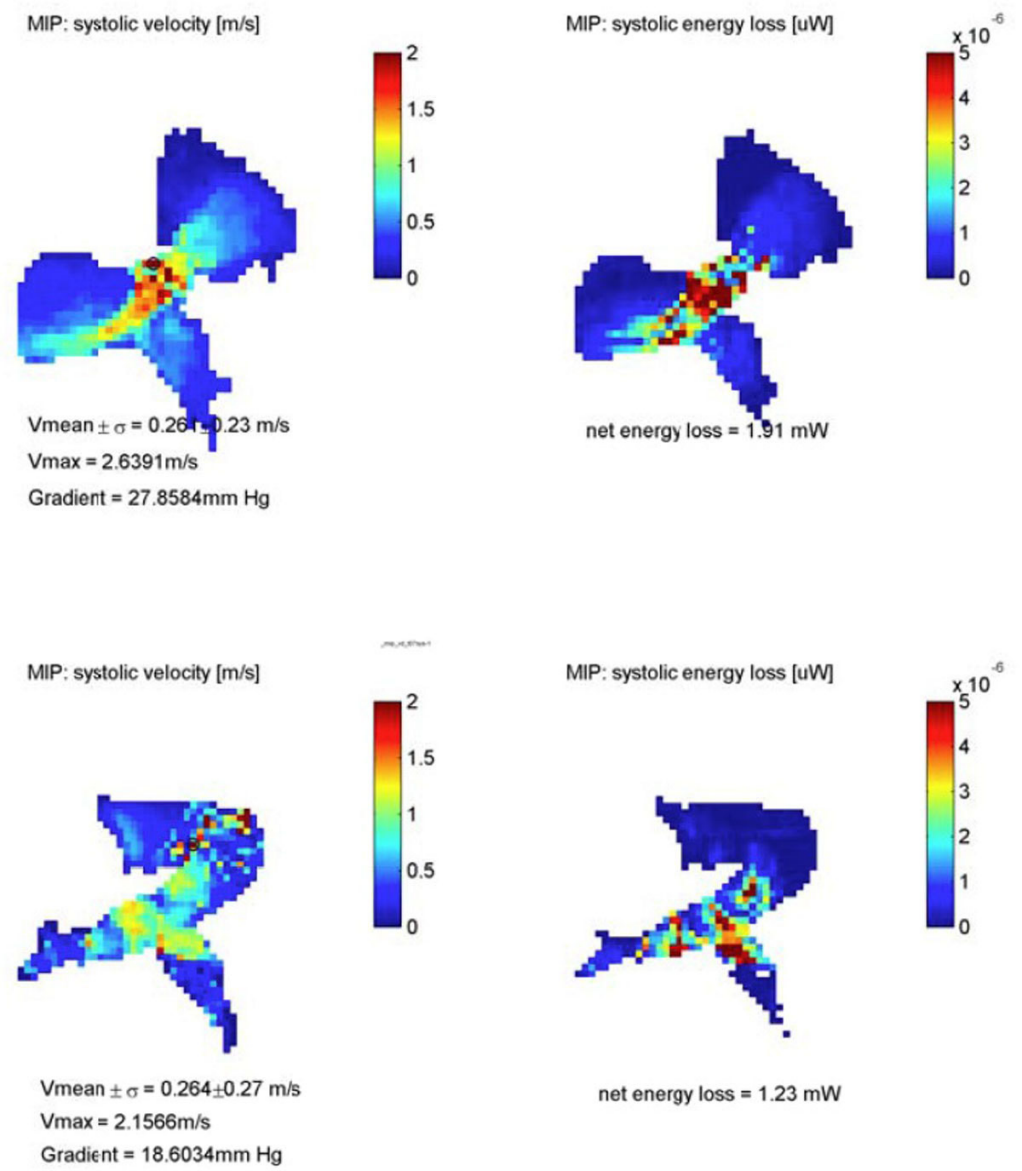
**Maximum intensity projections of velocity encoded data generated from improved PA segmentations allow efficient and improved visualization of residual outflow tract obstruction**.

18 pts with TOF (mean age: 13.1 ± 6.7 years) underwent simultaneous 4D flow and 2D PC MRI. 2D PC studies (1 mm in-plane spatial res, 30 true phases/cardiac cycle temp res) of the aortic root (Ao), pulmonary trunk (PT), and right and left PAs were analyzed (Medis, Leiden, The Netherlands). Standard 4D flow (2.5 mm in-plane spatial res, 80 ms temp res) analysis included calculation of a 3D-PC-angio which was used to position analysis planes in the Ao, PT, LPA and RPA (EnSight 10, CEI, Apex, NC). To isolate vessels of interest, velocity data were further segmented into aortic and PA volumes using an enhanced workflow (Mimics, Materialise, Leuven, Belgium) and similar analysis planes applied. 4D and 2D PC net flow, regurgitant fractions and peak velocities were calculated from analysis planes. Peak velocities were also determined from the segmented PT volumes and displayed as maximal intensity projections (Figure 1) using a custom application. Comparison of 4D and 2D PC data was performed using Pearson's correlation coefficient (r).

## Results

There was good agreement between 4D flow and 2D PC MRI for quantification of pulmonary regurgitant fraction and net flow (r = 0.68, p = 0.007; r = 0.68, p = 0.001). Correlation for the same parameters was modestly improved using enhanced segmentation (r = 0.77, p = 0.002; r = 0.74, p = 0.002). Agreement was excellent for aortic net flow using both techniques (r = 0.92, p < 0.001 for 2D vs 4D; r = 0.88, p < 0.001 for 2D vs enhanced 4D). For PT peak velocities, a moderate relationship was found between 4D flow and 2D PC (r = 0.69, p = 0.008 for 2D vs 4D; r = 0.60, p = 0.008 for 2D vs enhanced 4D), but peak velocities by volumetric 4D analysis were significantly higher than 2D PC (mean 2.3 ± 0.6 vs 1.4 ± 0.4 m/s, p = 0.01).

## Conclusions

4D flow MRI quantification showed good correlation for flow parameters used to characterize TOF with pulmonary regurgitation, including net flow and regurgitant fraction. Enhanced segmentation facilitates easier placement of analysis planes and may improve quantification. Improved segmentation facilitates visualization of residual right ventricular outflow tract obstruction but further comparison of peak velocity data is needed.

## Funding

NIH/NHLBI 1R01HL115828-01.

